# Observations on the Effects of Magnesia, in Preventing an Increased Formation of Uric Acid; with Some Remarks on the Composition of the Urine

**Published:** 1810-09

**Authors:** William T. Brande

**Affiliations:** the Society for the Improvement of Animal Chemistry, and by them to the Royal Society


### *Observations on the Effects of Magnesia, in preventing an increased Formation of Uric Acid; with some Remarks on the Composition of the Urine.* Communicated by Mr. William T. Brande, F. R. S. to the Society for the Improvement of Animal Chemistry, and by them to the Royal Society.

( From the Philosophical Transactions for 1810, Part I. )

Mr. Home's inquiries into the functions of the stomach, and his discovery of liquids passing from the cardiac porton into the circulation of the blood, led him to consider, that the generality of calculous complaints might possibly be prevented, by introducing into the stomach, such substances as are capable of preventing the formation of uric acid, and that this mode of treatment would have many advantages over the usual method, which consists in attempting to dissolve the uric acid after it is formed.

He consulted Mr. Hatchett on the substance most likely to produce this effect, and asked if magnesia, from its insolubility in water, was not well adapted for the purpose, as it would remain in the stomach, until it should combine with any acid, or be carried along with the food towards the pylorus.

Mr. Hatchett knew of nothing more likely to produce the desired effect; and on putting this theory to the test of experiment, it was found by a very careful examination of the urine, that in several instances where there was an increased formation of uric acid, magnesia diminished it in a much greater degree than had been effected by the use, and that a very liberal one, of the alkalies in the same patient.

This circumstance led Mr. Home to wish for a more complete investigation of the subject, and he requested me me to assist him in the prosecution of it. Since that time many opportunities have occurred of carrying on the inquiry during an attendance on patients labouring under calculous complaints.

It is proposed to lay the results of our joint labours before this Society, with a view to establish a fact of so much importance in the treatment of those diseases.

The four following cases include the principal varieties of the disorder, which have been met with, and are therefore selected from among many others, to prevent unnecessary repetitions. In each of them the urine was occasionally carefully analysed.

Case I. A gentleman, sixty years of age, who had been in the habit of indulging in the free use of acid liquors, had repeatedly passed small calculi composed entirely of uric acid; his urine immediately after being voided, deposited at all times a considerable quantitiy of that substance, in the form of a red powder, and occasionally in large crystals.

Nine drachms of subcarbonate of soda, dissolved in water highly impregnated with carbonic acid, and taken in the course of the day at three doses, appeared to have no effect whatever on the formation of uric acid; the red sand was deposited as usual, and the small calculi continued to form.

On account of the inefficacy of this medicine, he was advised to try the vegetable alkali, and three drachms of subcarbonate of potash, dissolved in water slightly impregnated with carbonic acid, were taken at similar intervals.

The deposit of uric acid in the urine was now somewhat diminished; but during this free use of alkalies, which, with little interruption, was persevered in for more than a year, the small calculi still continued to be voided.

The very unusual disposition to form uric acid, and the complete failure of the common alkaline medicines, rendered this case particularly favourable for the trial of magnesia, as it would afford an opportunity of comparing its effects with those of the alkalies.

Previous to giving the magnesia, the urine was examined, to ascertain the quantity of uric acid it contained; this being done, the patient was directed to take fifteen grains of magnesia three times a day, in an ounce and a half of infusion of gentian: in a week the uric acid was found, by examining the urine, to have diminished in quantity, and after the first three weeks it was only occasionally met with.

The

The use of magnesia has been persevered in for eight months, during which time no calculi have been voided, nor has there been any material deposit in the urine.

This patient was extremely subject to heartburn, and he likewise complained of a sense of weight and uneasiness about the region of the stomach, both of which symptoms have disappeared.

Case II. A gentleman, about 40 years of age, had during four years occasionally voided considerable quantities of uric acid, in the form of red sand, and had once passed a small calculus.

His urine was generally more or less turbid, and after taking any thing which disagreed with his stomach, even in a slight degree, the red sand often made its appearance. He had never used the alkalies nor any other medicine, to alleviate his disorder: he was consequently desired to take a drachm and a half of subcarbonate of soda, dissolved in a pint and a half of water highly impregnated with carbonic acid, in the course of the day, and to persevere in this treatment for some time.

On the 30th of January 1809, he left London, and returned on the 6th of March following.

During his absence he had voided rather less uric acid than usual, but had had one severe attack, in consequence of which, twenty drops of the solution of pure potash were added to each dose of the soda water: this, however, had not the desired effect; for on the 10th of March, having taken more wine than usual on the preceding day, he was attacked with pain in the right kidney, and voided with his urine a considerable quantity of uric acid, in the form of minute red crystals. During the succeeding day, he made but little water, which deposited a copious sediment of red sand.

For the removal of this symptom, he was directed to take magnesia, in the dose of twenty grains every night and morning, in a little water; for three successive days his bowels were unusually relaxed, but afterwards became regular. He persevered in its use for six weeks without intermission; his urine was several times examined during that period, and contained no superabundant uric acid; and he has not had the slightest return of his complaint, although he has put himself under no unusual restraint in his mode of living.

Case III. About the middle of October 1808, a gentleman, 43 years of age, after taking violent horse exercise, was seized with pain in the right kidney and ureter. In the the course of the night he passed a small uric calculus. For some months previous to this attack, he had felt occasional pain in the kidney, but had never voided either calculi or sand. His urine was now always turbid, and occasionally deposited red sand.

On the 28th of October he began the use of soda water, and for a time his urine was much improved in appearance, but the uric acid gradually returned, and at the end of December, notwithsanding the continued use of the soda water, he voided more sand, and his urine was more loaded with mucus, than it had ever been before.

In consequence of these symptoms, on the 3d of January 1809, he was directed to take twenty grains of magnesia every night.

The urine was examined after the third dose, and the deposit of red sand was diminished in quantity, but it did not disappear entirely, after the magnesia had been taken for three weeks.

About this time (on the 26th of January) he caught cold, and his urine was again very turbid, but this was found to be wholly the effect of mucus, and the symptom soon left him.

On the 30th of January he took twenty grains of magnesia, and repeated it every night and morning, until the 1st of March, when his urine was perfectly healthy, and he left it off.

On the 1st of June he again voided a little uric acid, in the form of red crystaline sand; this attack was attended with a slight pain along the right ureter. He returned to the use of the magnesia, which he took twice a day for three weeks, in the same dose as before, and from that time to the middle of November there had been no symptoms of a return of the complaint.

Case IV. A gentleman, aged 56, after recovering from a severe fit of the gout, voided constantly a large quantity of mucus in his urine, a symptom which he had never before noticed. There was also, occasionally, abundance of red sand, consisting principally of uric acid, but he had never voided a calculus.

His stomach was uncommonly weak, he was often affected with heart-burn, and an almost constant pain in the neighbourhood of the right kidney. He had been in the habit of taking tincture of bark, and other spirituous medicines, from a belief that the pain in his right side arose from gout in the stomach.

He had already attempted to use the alkalies, which had produced produced such unpleasant sensations in the stomach, that he could not be prevailed upon to try them again in any form.

Under these circumstances, he readily acceded to a new plan of treatment. He was directed to omit the use of spirituous medicines, and take twenty grains of magnesia three times a day in water; but this operating too powerfully upon the bowels, the same quantity of magnesia was taken twice a day only, with an addition of five drops of laudanum to each dose.

This plan was pursued without intermission for three weeks, and he received considerable benefit, as far as concerned the state of the stomach, and pain in the region of the kidney.. The urine, which was examined once a week, was also, on the whole, improved; but it occasionally deposited a very copious sediment, consisting of uric acid, with a variable proportion of mucous secretion.

After a further continuance of the use of the magneia, for three weeks, the urine was often much loaded with uric acid and mucus; but these appearances, which before the use of the magnesia were constant, are now only occasional, so that the disposition to form a redundant quantity of uric acid is much diminished: it is also deserving of remark, that there has not been the slightest symptom of gout from the time of the last attack, which is more than a year back, a longer interval of ease than this patient has experienced for the last six years.

He has now omitted the regular use of the magnesia; but on perceiving any unpleasant sensation in the stomach, he returns to it for a week or ten days, and then again leaves it off.

From the preceding cases it appears, that the effects of magnesia taken into the stomach, are in many respects different from those produced by the alkalies, in those patients in whom there is a disposition to form a superabundant quantity of uric acid.

With a view to ascertain their comparative effects on healthy urine, when taken under the same circumstances, the following experiments were made.

*Experiment* 1. *On Soda.*

Two drachms of subcarbonate of soda were taken on an empty stomach at nine o'clock in the morning, dissolved in three ounces of water, and immediately afterwards, a large cup of warm tea.

In

In six minutes, about one ounce of urine was voided; in twenty minutes six ounces more; and after two hours, a similar quantity.

The first portion became very turbid within ten minutes after it had been voided, and deposited a copious sediment of the phosphates, in consequence of the action of the alkali upon the urine. It slightly restored the blue colour to litmus paper reddened with vinegar: the alkali, therefore, was not merely in sufficient quantity to saturate the uncombined acid in the urine, and consequently to throw down the phosphates; but it was in excess, and the urine was voided alkaline.

The urine voided after twenty minutes, also deposited a cloud of the phosphates; but the transparency of that voided two hours after the alkali had been taken, was not disturbed.

Here, therefore, the effect of the alkali upon the urine was at its maximum, probably in less than a quarter of an hour after it had been taken into the stomach, and in less than two hours the whole of the alkali had passed off.

*Experiment 2. On Soda, with Excess of Carbonic Acid.*

The same quantity of soda, dissolved in eight ounces of water very highly impregnated with carbonic acid, was taken under the same circumstances as in the former experiment, and the urine was voided at nearly similar intervals.

The separation of the phosphates was less distinct, and less rapid. In two hours after the urine had been voided, there was a small deposit, composed principally of phosphate of lime; there was also a distinct pellicle on the surface, consisting of the triple phosphate of ammonia and magnesia. This appearance produced by the escape of the carbonic acid, which had before retained the ammoniaco-magnesian phosphate in solution, and which now occasions its depositions on the surface, is by no means uncommon, even in the urine of healthy persons: in the present instance, it appears to prove, that carbonic acid passes off from the stomach by the kidneys; for, after taking the alkalies in water very highly impregnated with it, the pellicle is uniformly produced, and is also much more abundant and distinct than under any other circumstances.

In similar experiments with potash, the results were in all all cases as similar as could be expected in researches of this nature.

*Experiment 3. On Magnesia.*

Magnesia was taken under circumstances similar to those of the soda in the former experiment: in the quantity of half a drachm, it produced no sensible effect upon the urine during the whole day. When taken in the dose of a drachm at nine o'clock in the morning, the urine voided at twelve o'clock became slightly turbid: at three o'clock the effect of the magnesia was at its maximum, and a distinct separation of the phosphate took place, partly in the form of a film, which when examined was found to be the triple phosphate of ammonia and magnesia, and partly in the state of a white powder, consisting almost entirely of the triple phosphate and phosphate of lime.

The effect of large doses of magnesia, in producing a white sediment in the urine, is very commonly known, and has been erroneously attributed to the magnesia passing off by the kidneys.

These experiments show that magnesia, even in very large doses, neither produces so rapid an effect upon the urine, nor so copious a separation of the phosphates, as the alkalies; on this its value as a remedy in calculous disorders seems materially to depend.

*Experiment 4. On Lime.*

Two ounces of lime water, taken in the morning upon an empty stomach, with a cup of milk and water, produced no effect whatever.

A pint of lime water, taken at four intervals of an hour each, produced a slight deposition of the phosphates at the end of the fifth hour. The urine voided at the third hour was not at all affected; at the fifth hour, the effect appeared at its height, but was not nearly so distinct as from small doses of soda, notwithstanding the insoluble compounds which lime might be expected to form with the acids in the urine.

The unpleasant taste of lime water, the quantity in which it requires to be taken, on account of the small proportion of the earth which is held in solution, and the uncertainty of its effect, are circumstances which render it of little use, excepting in some very rare cases, where it has been found to agree particularly well with the stomach.

The effect of carbonate of lime upon the urine was much less distinct than that of lime water; at times it produced no effect, but when taken in very large doses, a slight deposition of the phosphates was produced.

These experiments were repeated upon three different individuals, and there was always an uniformity in their results.

When the medicines were taken some hours after food being received into the stomach, their effects upon the urine were retarded, but not prevented.

The effects of many other substances upon the urine were examined into during this investigation; but they varied so much according to circumstances, that no satisfactory results were produced.

As it is found in the foregoing experiments, that the effects of soda on the urine are modified by the presence of carbonic acid, the following experiment was made, to ascertain whether any sensible effects are produced by that acid on healthy urine.

Twelve ounces of water very highly impregnated with carbonic acid, were taken upon an empty stomach at nine o'clock in the morning. At ten o'clock about eight ounces of urine were voided, which had a natural appearance, but, when compared with urine voided under common circumstances, was found to contain a superabundant quantity of carbonic acid: this gas was copiously given off when the urine was gently heated, or when it was exposed under the exhausted receiver of an air-pump.

In a patient who had a calculus of large dimensions extracted from the bladder, composed entirely of the phosphates, and whose stomach did not admit of the use of stronger acids, carbonic acid was given in water; it was found peculiarly grateful to the stomach, and upon examining the urine during its use, the phosphates were only voided in solution; but when at any time it was left off, they were voided in the form of white sand.

